# Rational Medicine: Its Position and Prospects

**Published:** 1861-03

**Authors:** 


ART. III.—1. *Rational Medicine: Its Position and Prospects.*—An Oration delivered before the members of the Hunterian Society, on the 15th February, 1860. By STEPHEN H. WARD, M.D., LOND., M.R.C.P., Physician to the Seaman's Hospital "Dreadnought," Vice-President of the Hunterian Society, etc. 8vo., pp. 52. London: John Churchill, 1860.

2. *Currents and Counter-Currents in Medical Science.*—An Address delivered before the Massachusetts Medical Society, at the Annual Meeting, May 30, 1860. By OLIVER WENDELL HOLMES, M.D. 8vo., pp. 48. Boston: Ticknor & Fields, 1860.

THE first address, at the head of this article, delivered on the forty-first Anniversary of the Hunterian Society, by its Vice-President, Dr. Stephen H. Ward, is an able and dignified exposition of the position and prospects of what the author is pleased to denominate *Rational Medicine.* The spirit in which it is written is highly commendable, for, while the author expresses his views clearly, and with a complete sense of the responsibility resting upon him in so doing, he deals kindly with his professional brethren, and cordially acknowledges the value of their investigations and labors for the amelioration of disease; so that, although we are unwilling to go quite as far as he does in our ideas of general expectant treatment, we cannot but feel that his intentions are good, and his love for the profession of his choice unabated.

His position is thus defined:—

"Under the term *medicine*, I embrace its different branches, and the art as well as the science; and I call that *rational* medicine which has its foundations laid in a recognition of Nature's resources in disease as well as in health; which feels that its object is science, not mystery; which, for its advancement, has recourse to philosophical appliances and methods of investigation; which acknowledges no means but such as are adequate to ends; which holds hypotheses upon uncertain tenure, ready to relinquish them as fresh compelling facts flow in; and which, eminently eclectic, avails itself of what is good in all systems, and is yet slave to none!"

No position could be more satisfactorily explained. This is precisely the ground occupied by every scientific physician, and all such practice upon the rational method; we therefore sincerely deprecate the attempt to force upon us the belief that an old idea is new—that the *vis medicatrix naturæ* has just been discovered. We would be better satisfied did our author simply attempt to show that in our over-anxiety to master disease, many, perhaps most of us, have partially lost sight of the fact that we are the *servants*, not the masters of Nature, and *tend* to err on the side of over-medication. That unscientific and ill-informed practitioners should be polypharmacists and over-dosers is not strange when the cry of the sufferer is for immediate and visible means of relief; but that these instances should be permitted to reflect discredit on the established principles of medicine, is unfair in the extreme.

We also object strongly to the statement that medicine "avails itself of what is good in all systems,"—that is, if the various irregular practices of the day are referred to; but we cannot think that our author intended this construction; yet, as this assertion without limitation is sometimes made by others, we take occasion to deny that we go abroad either to hydropathy, homœopathy, or any other pathy, to cull and borrow. Whatever is good in these systems has been taken from medicine proper as a basis for the excrescences which constitute them, and after the chaff has been blown away, nothing remains but that which belongs to legitimate science. The fountain does not look to the turbid stream to vivify its crystal waters, and the rain drops which replenish it, yield up its own, and that alone.

"A consideration of the position and prospects of rational medicine," it is stated, "resolves itself into the causes affecting such; and these may be arranged under two heads, *philosophical* and *ethical*."

Under the *philosophical* causes, he speaks of the use of the speculum and stethoscope, of microscopy and chemistry, and of the application of logic to medical inquiry, treating, as parts of this, of observation, the numerical method, and hypotheses.

Under the *ethical* causes, he enumerates, the spirit in which it is prosecuted, the conduct of the members of the profession toward one another, and considerations of a political and social nature.

In the consideration of the *philosophical causes*, while our author acknowledges, and bears a just tribute to, the usefulness of the speculum and stethoscope, the microscope and chemical agents in the hands of able workers, in greatly enlarging and strengthening the scientific basis of medicine, notwithstanding the subtle character of the phenomena of life both in health and disease, yet he is assured that the very instruments by which advancement has been mainly effected contain within themselves the elements of abuse. And he thinks that we are too ready to exchange the old and tried for the more fascinating new lamps, and to be attracted by that which is tangible, which speaks directly and speciously to the mind through sight, hearing, and touch, conveying a present sense of power, rather than by the larger contemplation of complex phenomena, and the postponed conclusions it entails. And his effort is to show that these agents have been abused, not only in their improper use, but in the attempt to set up exclusive claims for them as individual arbiters of disease, whereas they should occupy entirely subordinate positions in their influence upon medicine.

He appreciates the value of the stethoscope and speculum, and simply refers to the abuse of the latter in unprincipled hands. Concerning the microscope, while duly according it high rank among the means of advancing anatomy, physiology, pathology, diagnosis, and medico-legal inquiry, he thinks that "too much time is being devoted to it by the school of young medicine," and says truly that undue devotion to this means of inquiry induces a narrow view of disease and treatment, occupying much time to the exclusion of higher considerations. In regard to chemistry, he gives it the credit of determining the composition of the solids and the fluids of the body, of aiding clinical medicine by analyses of excreted fluids, and in special therapeutics of substituting active and elegant, for crude and bulky preparations; but with great force remarks: "I cannot, however, admit the higher pretensions which some chemists have claimed for their science in reference to physiology, pathology, and therapeutics; the pretension of explaining the phenomena of sciences in which vitality plays the prominent part, or of predicating from the results of laboratory experiments, what should take place in the living organism." He then illustrates his meaning by showing that observation and experience have proved the food theory of Liebig to be, in great degree, erroneous; that alcohol is not simply a heat-making fluid, but, besides being an arterial and nervous stimulant, it doubtless, by healthful use, prevents the waste of the tissues, and thus becomes a substitute for a given amount of food. He also shows that the attempt to explain the action of drugs on purely chemical grounds has failed, since, instead of observing an operation in a clean retort affording complete isolation from disturbing influences, our calculations are affected by the presence of nervous force and the inherent vitality of the receptacle. Drugs therefore act, not upon chemical grounds alone, but by influences other than those based upon chemical reactions; thus nitrate of potash, which has been thought to act in acute rheumatism by solving the fibrin which is in excess, by its known power of reducing the action of the heart, must no less aid the curative process.

Again, the author asserts that he has no desire to detract from the merits of microscopy or chemistry. He says:—

"I only desire to protest against their exerting an undue influence, and absorbing too exclusively our time. I wish to respect the study and laboratory as important adjuncts to the chamber or the ward, not, as they are being made by some men of the rising school, the principal theatre of the medical man's researches. It should ever be remembered, that in disease as in health we have to deal with that principle which proclaims its presence in countless phenomena of life, but which the keen scalpel of the anatomist, the powerful object-glass of the microscopist, the subtle test of the chemist, must ever fail to detect."

*Observation*, being at the basis of all reasoning, we are properly told, should be exact and comprehensive; and that many of the contradictory experiences of medical men are owing to incorrect and defective observation—that improved habits in this particular are required; besides which the want of attention to the larger phenomena of disease leading to confidence in prognosis, and to the recognition of crises and periodicity, is deplored.

The *numerical method* is believed not to admit of a very liberal application to medicine, as it is only of use in determining general facts, since many apparently similar cases differ materially in particulars, the result of modifying influences producing complex phenomena.

*Hypotheses* are held to be absolutely essential to science, as they are but steps in the progress to something more certain; but they must be built upon a sufficient basis—not being mere arbitrary conjectures; and are to be relinquished as fresh facts compel. Still, the usual steps in the process of reasoning are to be carefully employed to arrive at principles of much higher value than those based on mere empiricism.

The author believes that the philosophical appliances and the philosophical method of investigation to which he has alluded, have done much for the science of medicine, but far less for the art, in which "the labor has truly ever been in a circle, the alternating links of which have been disease and drugs." The cause of this difficulty, he thinks, is that professors of the healing art have never taken, "as the basis of their reasoning, the curative resources of Nature herself, as ascertained by study of the natural course of disease," and that consequently all their ratiocinations upon the actions of medicines have been founded on the comparison of one method of treatment with another; and that this is the main cause of "much of that uncertainty which has been the scandal and reproach of our art."

He believes that if the proper confidence in the inherent restorative powers of the constitution were felt, acknowledged, and acted upon, cures would not be, as they often are, falsely attributed to remedies on the *post hoc ergo propter hoc* principle, which is the great argument in support of quackery, backed up by the system of polypharmacy of orthodox practitioners, "which has weakened their position in regard to remedies where they are undeniably beneficial, and detracted from the credit which has ever been justly their due, of having been alive to the importance in the treatment of disease of modified hygienic measures."

"A conviction of the large powers of Nature, and the comparatively limited powers of art, in the cure of disease," he says, "is, I am satisfied, daily gaining ground;" and he adduces in support of his position the views of Sir J. Forbes, who insisted upon the necessity of obtaining a knowledge of the course of diseases unbiased by special therapeutical remedies;—of Dr. Hughes Bennett, who insists upon our reading the book of Nature for ourselves, no longer led by the comparatively blind guides of the past, but with the light of advanced knowledge which has united physiology and pathology by showing, in regard to the laws of nutrition, that what is generally regarded as morbid is but healthy action modified to suit altered circumstances, deducing therefrom that we must economize, not depress the reparative powers;—of Dr. Todd, who enunciated almost similar pathological views, deducing hyper-stimulation as treatment, (with which, however, Dr. Ward does not agree);—and his own experience in the Dreadnought Hospital, and in his practice in fevers, dysentery, and other diseases.

To show "that a large number of diseases will do perfectly well without, and cannot be curtailed by, any special interference of art," he instances the exanthemata and fevers, in all of which nature sets up a process with a view of expelling the poison introduced from without. "In such diseases little is to be done beyond studying their natural course, and carrying out indications." For such he objects to the uniform adoption of a special plan of treatment for certain complications, as laid down in systematic treatises, and, in support of this view, refers more particularly to typhoid fever, in which he has observed, in cases uninfluenced by any special treatment, that the diarrhœa, especially in the absence of much eruption, is the principal part of the effort of Nature to eliminate the poison, and as such should not be unduly interfered with, under penalty of danger. His view of the treatment of such cases seems to be summed up as follows:—

"I would add my protest against undue interference in fevers or the exanthemata, either in the way of stimulation or elimination, for Nature generally admirably adapts her operations to the powers of the patient. When she is unequal to her task, the assistance of the medical practitioner will, of course, be required."—

In accordance with which, he has previously stated that, in enteric fever, "should the evacuations be copious and exhausting, astringents and opium may be necessary," and subsequently, that notwithstanding the unaided powers of the system are frequently adequate to the repair of formidable dysenteric lesions under favorable hygienic conditions, rest, etc., yet he has met with cases which only yielded to the cautious and protracted administration of mercury.

"Having thus alluded to some diseases which, as a rule, the conservative powers implanted in the system are quite adequate to remove," the orator thinks it but right that he "should glance at others in which the assistance of art is more or less indispensable." He has found quinine necessary in ague, iodide of potassium in tertiary syphilis, iron in anæmia, cod-liver oil in struma, certain drugs to give mechanical relief in dropsy, opium and other anodynes as palliatives, and acknowledges that the rational co-operation of special medicines is required in many instances in which hygienic arrangements are alone inadequate.

Thus fully admitting the value of special therapeutics in particular cases, we are exhorted to abandon our position upon those points where weak and indefensible, and, by testifying to our reliance upon general therapeutics, strengthen ourselves in special matters where the ground is tenable, while we are assured that by a full recognition of Nature's resources in disease, medicine will make a more rapid and healthier progress, and that the practitioner "will be sustained by the conviction that his services will be as much needed as ever in his capacity of '*Naturæ minister.*'"

In the consideration of the *ethical causes* which influence the position and prospects of rational medicine, the author, with true dignity, avoids any attack upon quackery, but reprehends "any undue grasping after, or indirect means had recourse to of obtaining, practice; any attempt to make capital out of the unavoidable errors of others; or habitual exaggeration of cases and cures;" and asks us to consider whether this exists among us, great or humble, notwithstanding that men of the right spirit are undoubtedly with us.

He insists on the necessity of a uniform high standard of qualification being adopted by all examining boards, and alludes to the injurious operation of local measures upon the profession in England. Among these is the closure to medical men of the higher posts of honor, such as the legislature and the peerage, which incite members of other professions to intellectual effort. Besides which the colonial administrative offices, where medical experience is needed, not being, as a rule, filled by men most competent to speak on the subject of health, must be injurious to the prospect of the advancement of rational medicine, as also must be the method of electing medical men to offices under poor-law boards, in private charities, etc., by those who are in most instances entirely incompetent to judge of the respective merits of the candidates, or to appreciate the value of services rendered. And in truth many of these difficulties in England are not foreign to the United States.

Lastly, he says:—

"Rational medicine builds its expectations upon no one thing so much as upon the extension of education. It would fain see knowledge, and especially a knowledge of the natural sciences, diffused through all classes of the community." And further, "I am quite alive to the importance of instruction in the dead languages, but I desire to see some of the time devoted to them, set apart for the study of the living forms and actions of Nature, and of the laws which regulate organic life. At present there prevails all but complete ignorance of natural science, not only among the lower, but among the higher and in other respects, more educated classes. Such study has never formed part of the scheme of education at our foundation schools and older universities. It is this ignorance of Nature's laws, and especially of those which regulate life, which is the keystone to the fact that various forms of quackery are embraced by our statesmen, literary men, and by members of other learned professions; and that men give their judgment with ready confidence upon medical questions, who would think it the height of assurance for any unfurnished individual to pronounce upon their own avocations."

Without doubt, in this matter, he is clearly in the right.

In conclusion, he yields a glowing tribute to medical men for the large services which they have rendered the public in times of disease, pestilence, and death, on the battle-field and in the beleaguered city, and for their love of enterprise and scientific research, which has made many famous in the annals of every department of exact science, by the same method of philosophical investigation employed in medicine, where success has been but partial from the very nature of the case; and declares that both philosophically and ethically they have proved true to the best interests of their profession.

We have read this address with great satisfaction, because it contains much good, sound sense, and the views presented are for the most part well timed and kindly enforced; but we opine that the Doctor has *not* proved *conclusively* what he evidently set out to accomplish,—that Nature, or the inherent conservative power of the system, is adequate, in the *majority* of instances, to a cure, without the aid of art. It is true, he has shown us that certain fevers and exanthems having a recognized course, can only be watched, and untoward symptoms treated as they arise; that certain dysenteries and pneumonias are well managed by hygienic means alone, etc.; but he does not claim universal success by trusting to Nature entirely, even in these, nor do we find him selecting examples of cholera, pseudo-membranous croup, diphtheria, typhus, or yellow fever, to prove that recovery will in general be the result when the disease is subjected to favorable hygienic conditions alone. He has only shown, with fresh force, that in the treatment of disease we must *fully* recognize the *vis medicatrix naturæ*—that this is the basis upon which all our hopes of success must depend—that the laws of Nature in disease must be respected, and all our effects, to be useful, must be produced in accordance therewith—that there has been, and is, a *tendency* to forget these facts, and to place an undue reliance on special diagnostic means and drugs. We do not perceive that anything else has been shown, or that a startling revolution in the practice of medicine will be likely to follow.

We have gone thus extensively into the consideration of this pamphlet for the purpose of contrasting with it another, under a different title, but really on the same subject, and which appeared a short time subsequently, viz.:—

The address on Currents and Counter-Currents in Medical Science, delivered by Oliver Wendell Holmes, M.D., Professor of Anatomy and Physiology in the Massachusetts Medical College, a branch of Harvard University. This is perhaps one of the most curious productions among the fugitive medical literature of the day to which our attention has been directed. When we opened it for perusal, we certainly expected to find no very great excess of medical faith, for medical experience does not seem to be in the author's peculiar line, but we were not prepared for a sweeping tirade against the profession as a body, with insinuations more or less direct, concerning the probity of its members, coming from an individual who has been actively employed in fitting many of them for their vocation, and who had been so honored by his general associates as to be placed in perhaps the highest position in their gift. This position he has with peculiar indelicacy taken advantage of to castigate them unmeritedly and unmercifully, so that we are not at all surprised to find, at a meeting of the Massachusetts Medical Society, subsequent to that upon which this much-to-be-deplored speech was delivered before them, that it was

"*Resolved*, That the Society disclaim all responsibility for the sentiments contained in this Annual Address."

Our author commences with a eulogy on the professional dead who departed during the interval of the Society's annual meetings. He tells how near the physician comes to the hearts of the people, how he is the *main reliance* in sickness, how he is valued when living, and how his memory is cherished when dead; and we would believe him sincere, did not his subsequent writing prove his first paragraph, viz.:—

"Our annual meeting never fails to teach us at least one lesson. The Art whose province it is to heal and to save, cannot protect its own ranks from the inroads of disease and the waste of the destroyer"—

To be the medium of a concealed sneer, and that his opening tribute to his brethren does not accord in any sense with his estimate of the value of the means which they employ, since their lives must be, according to his own showing, one vast waste of energy, an eternal labor in the wrong direction, one vast round of error and deceit.

The incompetency of medical art is consequently the burden of the discourse, the heads of which we shall directly present. It is firstly asserted that

A medical man, after twenty years of practice, is apt to suppose that he treats his patients according to the teachings of his experience,—that this may be true to some extent, but that it can be easily shown that the prescriptions of even wise physicians are very commonly founded on something quite different, such as fashionable special remedies, or popular theories which will be unrecognized at a future period, and only viewed as a fashion of the by-gone time,—that there are in every calling those denominating themselves *practical men*, who go about the work of the day before them according to the rules of their craft, asking no questions of the past or of the future, and who, pulling the oars of society, have no leisure to watch the currents which carry them this and that way, rendering their work worse than useless unless they get out of the wrong and into the right ones, so that, if they look not up and around, they are carried to ends little dreamed of, as is the case with the physician who, calling himself a practical man, refuses to recognize the larger laws which govern his changing practice.

"The truth," he goes on to say, "is, that medicine, professedly founded on observation, is as sensitive to outside influences, political, religious, philosophical, imaginative, as is the barometer to the changes of atmospheric density." And, in support of this statement, he proceeds to "observe the coincidences between great political and intellectual periods, and the appearance of illustrious medical reformers and teachers," from the age of Plato and Hippocrates to the time of Benjamin Rush, who, he believes, was the intellectual offspring of the movement which produced the American Revolution.

That medical men of twenty years' experience should be *apt* to suppose that they treat their patients according to the teachings of experience, is not strange, and we think it not unlikely that their view of the matter will prove, as a rule, to be correct, Dr. Holmes to the contrary notwithstanding, since men of any judgment, especially teachers and leaders in medicine, found their practice upon accumulated observations, and whatever hypotheses are formed as a basis for their theories, result from such observations; these, however, may be incomplete or influenced by disturbing causes, which, after a time, being discovered, lead to new observations, the eliciting of fresh facts, a consequent modification of theory and improved practice. Essential changes, therefore, at the present day, in the treatment of disease, do not relate to fashion, either in theory or in practice, but depend upon the experience of the wise physician. It is certainly true that he who fails to recognize the larger laws of disease retrogrades while he seems to advance, but such a one is simply a practitioner, not a physician. That the impetus to the prosecution of medicine is influenced by the predominant tendency of the age, is a point which, perhaps, none will care to dispute; but that its sensitiveness to the outside influences enumerated is as delicate as that of the barometer may be considered as a poetic license of the widest latitude.

With this exposition of his general views, our author introduces us to the "main intellectual current of the time," and points out "some of the eddies which tend to keep the science and art of medicine from moving with it, or even to carry them backwards."

After some little difficulty, from the peculiarly loose and rambling character of the discourse, we have extracted the following heads:—

"We who are on the side of 'Nature,' please ourselves with the idea, that we are in the great current in which the true intelligence of the time is moving."—

Nature, being previously and afterward described to be, trust in the reaction of the living system against ordinary normal impressions, as opposed to Art, or *professional tradition.*

The eddies or counter-currents are—

Confidence in the use of drugs, resulting in over-medication;
Natural incapacity for sound observation;
Inability to weigh the value of testimony;
Inveterate logical errors;
Outside pressure of dose-loving public;
Old superstitions and false theories;
The practice of making a profit on the medicine ordered;
Certain special American influences;
Medical journals which must find wonderful cures to fill their pages;
Teachers in medical schools, in cities and wandering over the country, claiming as much as possible for our art.

These latter, then, are some of the counter-currents which, we are told, are *hindering* the onward progress of medicine, "that withered branch of science," as we are reminded it was called at a meeting of the British Association, which, Dr. Holmes informs us, it is so painful (!) for him to remember. And all these divisions are treated with an exaggeration and a flippancy entirely, we think, unbecoming one who lays claim to be considered a philosopher and a scholar.

We have a preliminary panegyric on Boston, in this wise: "If there is any State or city which might claim to be the American headquarters of the nature-trusting heresy, provided it be one, that State is Massachusetts, and that city is its capital." To this we might reply, in the same strain: In the name of the prophet, figs! while we assert that Boston is no more nature-trusting than other cities, and that the effect of such doctrines is only injurious to the profession by undermining the confidence of the public in it (as he suggests that some suppose) when advanced to the untenable extreme of the writer, who believes—

> That if every drug from the vegetable, animal, and mineral kingdom were to disappear from the market, physicians would exist, be required, and respected as now, whose province should be to guard against the causes of disease, to eliminate them if possible when still present, to so order the conditions of the patient that the system might right itself, to predict the course of disease and thereby calm the exaggerated fears of sufferers and their friends, or warn them in season of impending danger.

Most wonderful, therefore, would be the duties of physicians, who perhaps will be enabled to some extent to take this stand when the Millennium arrives, which will be, in all probability, the earliest period when the *causes* of disease will be absent, and consequently their effects; but until there is a complete change in the condition of human affairs, causes *will* exist to produce disease, which "Nature," unaided by Art, cannot carry to successful issue.

We would be pleased to discover where the power of unaided Nature lies, notwithstanding its tendency, to cure cholera asphyxia, violent cholera morbus, syphilis, intermittent fever, and a host of other ills that flesh is heir to. How would the author himself be pleased should he unfortunately suffer with cholera, arising from some obscure cause, with the attentions of a physician, who, having no faith in drugs, should calmly make his case a study for the natural history of disease, contenting himself by so ordering the patient's condition that the system might right itself? Does he not see that the employment of various remedies, including drugs, has arisen from the very failure of unaided Nature to restore, or from a certainty that by their judicious use, the powers of Nature are so guided or quickened as to cut short the course of the disorder? We confess a decided partiality to drugs when used and not abused, and believe that faulty observation, inability to weigh the value of testimony, the enumeration of successful cases only, the *post hoc ergo propter hoc* error, and the assumption of falsehood for fact and reasoning thereupon, which he touches upon as the ordinary modes of misunderstanding or misapplying the evidence of nature, are only the errors common to all investigations, and which do not lie against us nearly so much as we would be made to believe. The public, it is true, "insists on being poisoned," as is proved by the extensive patronage which they give to nostrums; but we do not think that the enlightened profession is hurried into extreme activity by it, nor do we think that this is the result of the superstition "that disease is a malignant agency or entity to be driven out of the body by offensive substances," or of "the detestable old superstitious presumption in favor of whatever is nauseous and noxious being good for the sick," still lingering in the minds of the public and the profession, but, of a well-attested observation, that Nature herself, in very many instances, unaided, is inadequate to a cure, together with an almost total want of knowledge on the part of the public, of either human structure or functions, which renders many an indiscriminating and easy prey to the specious advertisers who profit by their illness and ignorance.

As an example of the false theories which hinder the progress of medicine, the received notion of the condition of the tongue indicating in anywise that of the alimentary apparatus, is advanced as eminently striking, and we are indulged with the following highly philosophical remarks:—

"One practical hint may not be out of place here. It seems to be sometimes forgotten by those who must know the fact, that the tongue is very different, anatomically and physiologically, from the stomach. Its condition does not in the least imply that of the stomach, which is a very different structure, covered with a different kind of epithelium, and furnished with entirely different secretions. A silversmith will, for a dollar, make a small *hoe*, of solid silver, which will last for centuries, and will give a patient more comfort used for the removal of the accumulated epithelium and fungous growths, which constitute the 'fur,' than many a prescription with a split-footed ℞ before it, addressed to the parts out of reach."

In illustration, we are furnished with the wonderful history of the great prototype of this invaluable tongue-scraper, and told how, in conjunction with infusion of strawberry leaves and sassafras root, it *cured* a case of typhoid fever, thus saving the colony at Plymouth in 1623, through the gratitude of the patient, and thereby indirectly resulting in the formation of the Massachusetts Medical Society. All this, doubtless, shows the *hoe* to be a very wonderful instrument, which should, therefore, we think, occupy a conspicuous position on the seal of the society, but, it does *not*

show that the vascular system of the mucous membrane of the mouth and tongue is not continuously or sympathetically affected by morbid conditions of the same system in other parts of the alimentary mucous membrane, and that the resultant epithelial formations, although different in shape, cannot assume appearances which are indicative of trouble elsewhere.

The author deals with the practice of making a profit on the medicine ordered, as if it were a prevalent custom among physicians of the present day, and suggests, as a remedy, the abstraction of the cost of the drug from the consultation fee, instead of its addition. Now it is well known, that in all large cities, where the population is sufficient to warrant it, the business of compounding prescriptions is not in the hands of the prescriber, and that the Code of Ethics, binding upon all, expressly forbids collusion with the apothecary; while in the country, it is absolutely necessary for the physician to carry his own remedies and compound them, for which trouble he is hardly ever adequately remunerated. Our author, therefore, gratuitously insults the profession by directly charging on it sordid motives as influencing treatment.

The reference to other special American influences seems intended merely to introduce an attack upon Dr. Benjamin Rush, whose confidence in remedies, thereafter shaping American practice, is thus set forth:—

"But he could not help feeling as if Nature had been a good deal shaken by the Declaration of Independence, and that American art was getting to be rather too much for her,—especially as illustrated in his own practice," etc.—

And then, after a few elegant allusions to a people "which has *chewed the juice* out of all the superlatives in the language," asks—

"What wonder that the stars and stripes wave over doses of ninety grains of sulphate of quinine, and that the American eagle screams with delight to see three drachms of calomel given at a single mouthful?"

What professional man can fail to be delighted with language so choice, so poetic, so ennobling, especially if taken in connection with a statement on another page, where, in endeavoring to show that the search for remedies has been more anxiously pursued than the study of the causes of disease, we are told that—

"The very men who were discussing the treatment of the disease were stupidly conveying the infection from bed to bed, as *rat-killers* carry their poisons from one household to another."

In the anxiety to prove that attention to the obliteration of causes will do almost everything, it is said, with peculiar indelicacy, that if we do not succeed in this, we are to be—

"Reasonable and patient with Nature, who means well, but does not like to hurry, and who took nine calendar months, more or less, to every mother's son among us, before she thought he was fit to be shown to the public."

How our respect increases as our reading progresses!

We regret that our limits do not allow us to investigate much further the matter before us, for we should like to refer to the author's definitions of Nature, Art, Disease, Food, Medicine, and Physic, with which he paves the way to the subjoined propositions:—

1st. That disease and death are necessary in the present order of things.

2d. "The *presumption* always is that every noxious agent, including medicines proper, which hurts a well man, hurts a sick one."

3d. "Lastly, medication without insuring favorable hygienic conditions, is like amputation without ligatures."

Under the first head it is acknowledged that we must, from our imperfect intelligence, necessarily fail to keep the laws of the physical and spiritual universe, and that "Disease is one of the *penalties* of one of the forms of such failure;" and the author goes on to say:—

"But it is especially the *prerogative*, I had almost said *privilege*, of educated and domesticated beings, from *man* down to the *potato*, serving to teach them and such as train them, the laws of life, and to get rid of those who will not mind or cannot be kept subject to these laws."

Well done, Professor of Anatomy and Physiology! Is a potato then really an educated and domesticated being? And thus, potatoes, from their failure to keep these laws, must unite with men in possessing in one curious result, a penalty, a prerogative, and a privilege; certainly a most extraordinary combination.

And we are further led to infer that particular diseases must be blessings in disguise, since "the race would be ruined if art could ever learn always to preserve the individuals subject to them."

Under the second head, it is taken for granted that all noxious agents, that is, medicines proper, cost the patient five per cent. of his vital force, and then the profession is thus elegantly complimented:—

"In the game of Life-or-Death, *Rouge et Noir*, as played between the Doctor and the Sexton, this five per cent., this certain small injury entering into the chances, is clearly the sexton's perquisite for keeping the green table over which the game is played, and where he hoards up his gains."

In continuation, the professor's eminently philosophical views on the value of medicine are thus set forth:—

"Throw out opium, which the Creator himself seems to prescribe, for we often see the scarlet poppy growing in the cornfields, as if it were foreseen that wherever there is hunger to be fed there must also be pain to be soothed; throw out a few specifics which our art did not discover, and is hardly needed to apply; throw out wine which is a food, and the vapors which produce the miracle of anæsthesia, and I firmly believe that if the whole materia medica, *as now used*, could be sunk to the bottom of the sea, it would be all the better for mankind,—and all the worse for the fishes."

But why should we go further? We are already nauseated by the noxious stuff! We were led to believe, from the known literary ability of the orator, that we were to enjoy a pleasing discourse on the currents and counter-currents in medicine—we have risen from the perusal, convinced from many wonderful coincidences in headings, that Dr. Holmes, having had the advantage of reading the previous address of Dr. Ward, has appropriated his topics, and, transposing them, has employed them, unlike the latter in a delicate and philosophical manner to the purpose in hand, but as texts for an absurd and insulting tirade upon his Fellows, to which the severest reprobation would scarcely do justice. Not only, indeed, has he insulted the profession in general, but his immediate colleagues who are the teachers of materia medica and practice, obstetrics and surgery, cannot escape the blow. We wonder that he can remain any longer in communion with gentlemen, who, if they be true to the interests of science, must feel that he has belittled and disgraced it and them. We trust that this example will teach medical societies, all over the country, that when they appoint a man to address them upon their science and their art, it should be one who has *some* actual experience in the practice of his profession, and who is not a mere magazine writer and poet, as we fear has been the case in the present instance. The address of Dr. Holmes is in the worst possible taste, and we therefore regret that it was ever published, although we sincerely believe that in this instance the poison carries with it its own antidote.

